# Regulation of sterol metabolism by gut microbiota and its relevance to disease

**DOI:** 10.1080/19490976.2025.2589493

**Published:** 2025-11-26

**Authors:** Chaoyang Huang, Qingping Wu, Juan Wang, Jie Cai, Zelin Wang, Linghui You, Zhenjun Zhu, Yu Ding

**Affiliations:** a Department of Food Science and Engineering, College of Life Science and Technology, Jinan University, Guangzhou, Guangdong, People's Republic of China; b Institute of Microbiology, Guangdong Academy of sciences, State Key Laboratory of Applied Microbiology Southern China, Guangdong Provincial Key Laboratory of Microbial Safety and Health, Guangzhou, Guangdong, People's Republic of China; c College of Food Science, South China Agricultural University, Guangdong, Guangzhou, Guangdong, People's Republic of China

**Keywords:** Sterol metabolism, gut microbiota, metabolic diseases, dietary interventions

## Abstract

Sterols play an indispensable role in maintaining cell membrane stability, regulating hormone synthesis, and preserving physiological homeostasis. Recently, the function of the gut microbiota in modulating host sterol metabolism has become the focus of extensive research. Nonetheless, the specific functions carried out by the gut microbiota in sterol metabolism and their health implications remain unclear due to a lack of comprehensive synthesis and analysis. This review aims to consolidate current perspectives regarding the sources and metabolic mechanisms of sterols, with an emphasis on the involvement of gut microbiota in the biotransformation of zoosterols, phytosterols, and mycosterols. Additionally, it explores the pathological implications of sterol metabolism disorders in diseases such as Alzheimer’s disease and cancer. Finally, the review highlights the potential of dietary interventions to reshape gut microbiota composition and restore sterol metabolic homeostasis, presenting novel strategies for disease prevention and therapy through targeted modulation of sterol metabolism.

## Introduction

The intestinal microbiota, comprising bacteria, archaea, fungi, and protists, forms a highly intricate and dynamic ecosystem within the gut.^1^ These microbes possess extensive metabolic capabilities essential for maintaining microbiota balance and supporting host health and physiological functions.^2^ Their effects are mediated through direct cell-to-cell interactions and the production of bioactive metabolites, which are either synthesized by the microbiota or derived from host- and environment-derived molecules.

Sterols, a class of lipid compounds characterized by a tetracyclic cyclopenta-phenanthrene structure, are ubiquitous in animals, plants, and fungi. They play diverse biological roles in critical biological processes, including cell membrane integrity, signal transduction, hormone synthesis, and metabolic regulation.^3^^,^^4^ Based on their biological origin, sterols are generally categorized into three main groups: zoosterols, phytosterols, and mycosterols.^5^ Recent research has highlighted the complex relationship between the gut microbiota and sterol metabolism. Studies have shown that the gut microbiota not only modulates host sterol metabolism, particularly through cholesterol transformation, but also contributes to the metabolism of phytosterols and mycosterols. However, due to the limited integration and evaluation of existing findings, the precise role of the gut microbiota in sterol metabolism, and its implications for health and disease, remains to be fully elucidated.

In this context, this review systematically outlines the biological pathways involved in sterol metabolism, highlights the potential mechanisms through which the gut microbiota regulate these pathways, and emphasizes their roles in metabolism-associated chronic diseases, such as Alzheimer’s disease (AD) and cancer. Moreover, it explores the therapeutic potential of dietary interventions for modulating sterol metabolism, providing a theoretical foundation for translational research and clinical applications targeting microbiota-sterols interactions.

## Origin, metabolism, and their potential relationship of sterol

### Animal origin

**Figure 1.**
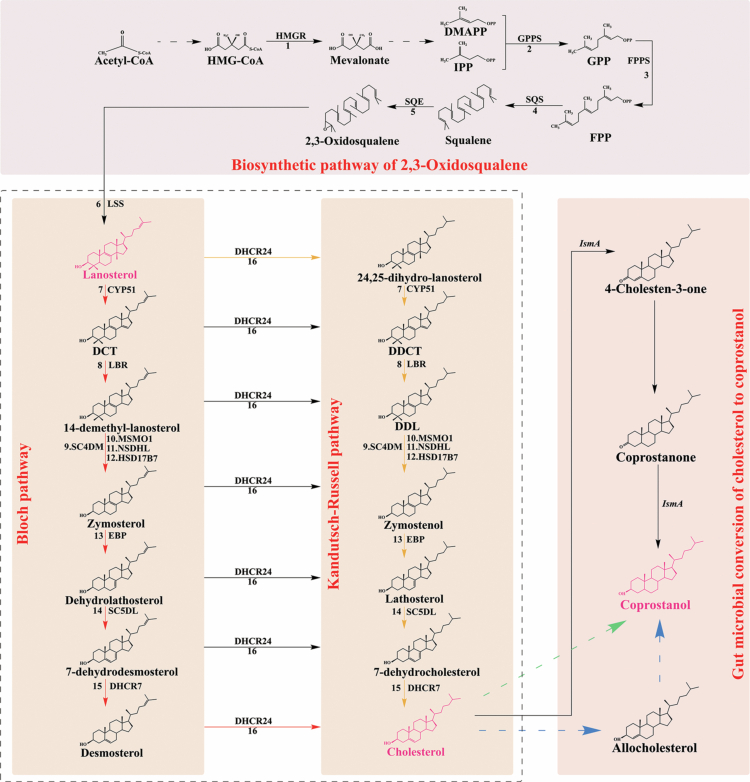
Transformation pathway of zoosterols. (i) 2,3-Oxidosqualene is synthesized from acetyl-CoA through a series of enzymatic reactions and is subsequently converted into lanosterol; (ii) Lanosterol serves as the precursor for cholesterol biosynthesis via two major pathways: the Bloch pathway (shown in red) and the Kandutsch-Russell pathway (shown in orange); (iii) Three pathways have been suggested for the conversion of cholesterol into coprostanol: (1) an indirect pathway via 4-cholesten-3-one and coprostanone (shown in black), (2) an alternative indirect route via allocholesterol (shown in blue), and (3) the direct reduction of cholesterol to coprostanol (shown in green). Confirmed enzymatic reactions are represented by solid lines, whereas hypothetical steps are indicated with dashed lines. Abbreviations: 1. HMGR, 3-hydroxy-3-methylglutaryl-CoA reductase; 2. GPPS, geranyl diphosphate synthase; 3. FPPS, farnesyl diphosphate synthase; 4. SQS, squalene synthase; 5. SQE, squalene epoxidase; 6. LSS, lanosterol synthase; 7. CYP51, sterol 14-demethylase; 8. LBR, δ(14)-sterol reductase; 9. SC4DM, sterol C-4 demethylation complex enzymes; 10. MSMO1, methylsterol monooxygenase1; 11. NSDHL, sterol-4α-carboxylate 3-dehydrogenase; 12. HSD17B7, 3-keto-steroid reductase; 13. EBP, C-8 sterol isomerasee; 14. SC5DL, sterol-C5-desaturase; 15. DHCR7, 7-dehydrocholesterolreductase; 16. DHCR24, δ(24)-sterol reductase; HMG-CoA, 3-hydroxy-3-methylglutaryl coenzyme A; IPP, isopentenyl pyrophosphate; DMAPP, dimethylallyl pyrophosphate; GPP, geranyl diphosphate; FPP, farnesyl pyrophosphate; DCT, 4,4-dimethyl-cholesta-8,14,24-trienol; DDCT, dihydro-4,4-dimethyl-cholesta-8,14,24-trienol; DDL, dihydro-14-dimethyl-lanosterol.

Zoosterols are a class of biomolecules characterized by a four-ring structure, with cholesterol being the primary representative.^6^ As a pivotal biological molecule, cholesterol maintains the structure and fluidity of cell membrane and serves as a precursor for steroid hormones, vitamin D, and bile acids (BAs).^7–9^ Other zoosterols include lanosterol and coprostanol. Lanosterol, a multi-ring sterol abundant in animal-derived substances like wool and sheep fat, exhibits a range of biological and pharmacological activities and has broad industrial applications.^10^ Coprostanol, on the other hand, is a microbial metabolite of cholesterol commonly found in animal feces and often used as a biomarker in ecological and behavioral studies.^11^ Structurally, cholesterol, lanosterol, and coprostanol share a conserved steroid core of three cyclohexane rings and one cyclopentane ring. Lanosterol closely resembles cholesterol but differs in side-chain configuration, whereas coprostanol features distinct modifications to both rings and side chains.^12^ From a biosynthetic perspective, lanosterol acts as an essential intermediate in the cholesterol synthesis pathway, undergoing a series of enzymatic transformations in humans to become cholesterol. In contrast, coprostanol is a terminal metabolic product of cholesterol, primarily formed through microbial reduction and excreted via bile.^13^ The dynamic interconversion and interaction among these sterols (Figure 1) help maintain a finely tuned internal balance that is essential for regulating physiological functions and supporting overall health.

Lanosterol, a tetracyclic compound, serves as the initial cyclic sterol intermediate in the cholesterol biosynthetic pathway and is essential for forming the cholestane ring structure.^14^ This process begins with acetyl-CoA entering the mevalonate (MVA) pathway, leading to the production of isopentenyl pyrophosphate (IPP) and dimethylallyl pyrophosphate (DMAPP), the fundamental building blocks for steroid biosynthesis. An essential enzyme in this pathway, 3-hydroxy-3-methylglutaryl coenzyme A (HMG-CoA) reductase (HMGR), catalyzes the rate-limiting step.^15^^,^^16^ Subsequently, IPP and DMAPP undergo condensation reactions catalyzed by geranyl diphosphate synthase (GPPS) and farnesyl diphosphate synthase (FPPS), resulting in the formation of geranyl diphosphate (GPP) and farnesyl pyrophosphate (FPP), respectively. Squalene synthase (SQS) then converts FPP into squalene (SQ), the universal precursor for all terpenoids and steroids.^17^ The pathway continues as squalene epoxidase oxidizes SQ to 2,3-oxidosqualene, which is subsequently cyclized by lanosterol synthase (LSS) to form lanosterol.^18^ As the first sterol with a complete tetracyclic structure, lanosterol serves as the pivotal branching point in cholesterol biosynthesis and forms the foundation for all steroid backbone synthesis.

During cholesterol biosynthesis from lanosterol, the pathway diverges into two routes: the Bloch pathway and the Kandutsch-Russell pathway, distinguished by the sequence of reactions involving δ(24)-sterol reductase (DHCR24).^19^ In the Bloch pathway, all intermediates from lanosterol to desmosterol retain a double bond at the C-24 position. The process begins with the C-14 demethylation of lanosterol by sterol C-14 demethylase, introducing a double bond. This is subsequently reduced by sterol C-14 reductase, forming 14-demethyl-lanosterol. The sterol C-4 demethylation enzyme system, including methylsterol monooxygenase, 4α-carboxysterol-3-dehydrogenase, and 3-ketosteroid reductase, removes two methyl groups from the fourth carbon to generate zymosterol.^20^^,^^21^ Zymosterol undergoes isomerization of the C-8 to C-7 double bond, catalyzed by sterol C-8 isomerase, followed by desaturation at the C-5(6) position via sterol C-5 desaturase, thereby producing 7-dehydrodesmosterol. This intermediate is then reduced at the C-7 double bond by 7-dehydrocholesterol reductase (DHCR7), leading to the production of desmosterol. Finally, DHCR24 reduces the C-24 double bond of desmosterol to generate cholesterol.^22^ Conversely, the Kandutsch-Russell pathway begins with DHCR24 converting lanosterol directly into 24,25-dihydrolanosterol, and all downstream intermediates, including 7-dehydrocholesterol (7-DHC), feature saturated side chains. Notably, any intermediate in the Bloch pathway can be transformed into its saturated counterpart through DHCR24 activity,^23^ highlighting the metabolic flexibility and interconnectivity between these two pathways.

Within the intestinal tract, cholesterol is metabolized into coprostanol through processes mediated by the gut microbiota. While this conversion primarily occurs in the colon, the presence of coprostanol in the proximal small intestine suggests that distinct microbial populations in both regions contribute to this conversion.^24^ Three biochemical pathways have been proposed for this conversion.^25^ The first involves 4-cholesten-3-one and coprostanone as metabolic intermediates. *Bacteroides dorei* D8, a strain isolated from human feces, utilizes this route.^26^ Similarly, *Eubacterium coprostanoligenes* (from pig wastewater lagoons)^27^ and *Eubacterium* ATCC 21408 (from rat feces)^28^ also employ related pathways, with the latter capable of isomerizing cholesterol to allocholesterol before reducing it to coprostanol. Apart from these indirect pathways, a direct process has also been proposed, in which the 5,6-double bond of cholesterol is directly reduced to form coprostanol without the formation of intermediate compounds.^28^ However, to date, no bacterial species has been definitively shown to perform this direct conversion.

### Plant origin

**Figure 2.**
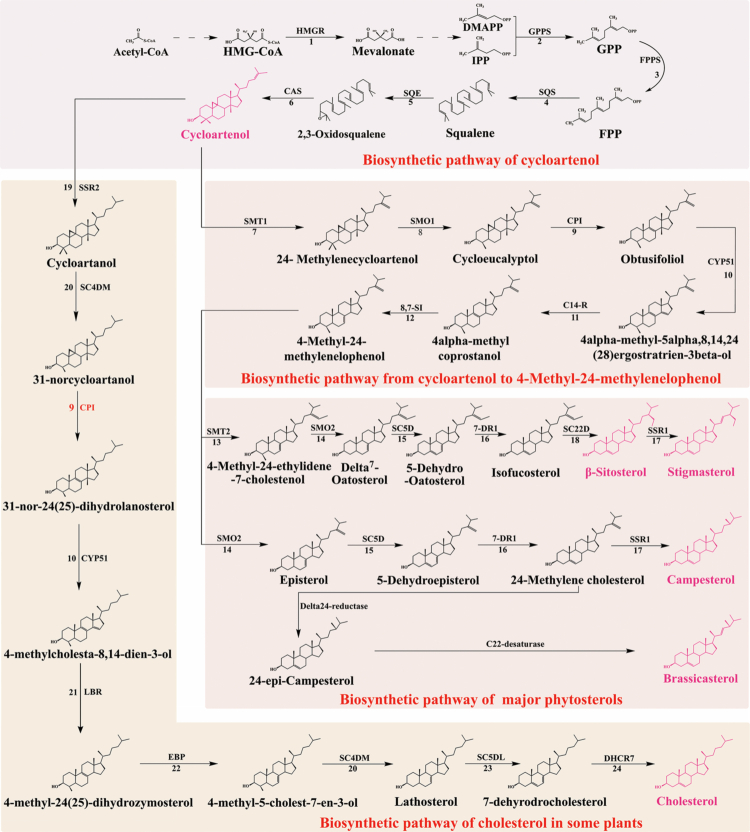
Transformation pathway of phytosterols. (i) 2,3-Oxidosqualene is synthesized from acetyl-CoA through a series of enzymatic reactions and subsequently converted into cycloartenol using cycloartenol as a key metabolic node; (ii) one branch of the metabolic flux toward the cholesterol biosynthesis pathway; and (iii) another branch is directed toward the 4-methyl-24-methylenelophenol, which serves as a precursor for (1) the formation of β-sitosterol and stigmasterol, (2) the biosynthesis of campesterol, and (3) the biosynthesis of brassicasterol. Abbreviations: 1. HMGR, 3-hydroxy-3-methylglutaryl-CoA reductase; 2. GPPS, geranyl diphosphatesynthase; 3. FPPS, farnesyl diphosphate synthase; 4. SQS, squalene synthase; 5. SQE, squalene epoxidase; 6. CAS, cycloartenol synthase; 7. SMT1, sterol C24-methyltransferase 1; 8. SMO1, sterol-4α-methyl oxidase 1; 9. CPI, cyclopropyl sterol isomerase; 10. CYP51, sterol C14-demethylase; 11. C14-R, sterol C14 reductase; 12. 8,7-SI, sterol δ8-δ7 isomerase; 13. SMT2, sterol C24-methyltransferase 2; 14. SMO2, sterol-4α-methyl oxidase 2; 15. SC5D, sterol C5-desaturase; 16. 7-DR1, sterol δ7-reductase; 17. SSR1, sterol side chain reductase 1; 18. SC22D, sterol C22-desaturase; 19. ERG4, C24(28) sterol reductase; 20. SC4DM, sterol C-4 demethylation complex enzymes; 21. LBR, δ(14)-sterol reductase; 22. EBP, C-8 sterol isomerase; 23. SC5DL, sterol-C5-desaturase; 24. DHCR7, 7-dehydrocholesterol reductase; HMG-CoA, 3-hydroxy-3-methylglutaryl coenzyme A; IPP, isopentenyl pyrophosphate; DMAPP, dimethylallyl pyrophosphate; GPP, geranyl diphosphate; FPP: farnesyl pyrophosphate.

Phytosterols, or plant sterols (PS), comprise a group of steroids characterized by a cyclopentane polyhydrophenanthrene backbone.^29^ They primarily exist in free and esterified forms within edible plant oils from nuts, grains, legumes, and seeds.^30–32^ Unlike plants, humans lack PS biosynthetic capacity, making dietary intake the exclusive source of PS in human blood and tissues.^33^ In plants, PS exist as either free sterols or conjugated sterols, with the latter further classified into sterol esters, sterol glycosides, and acylated sterol glycosides based on the groups attached to the C3 hydroxyl group.^34^ Structurally, PS share the characteristic cyclopentane polyhydrophenanthrene core but differ in their alkyl side chains at the C-17 position. A key distinguishing feature is the presence of either methyl or ethyl substituents at carbon 24, which forms the basis for their classification into C24-methylated sterols or C24-ethylated sterols.^35^ Despite their structural similarity to cholesterol, PS exhibit markedly different biological functions and follow distinct metabolic pathways, as shown in Figure 2.

Sterol biosynthesis in plants follows a distinct pathway from animals and fungi, utilizing cycloartenol rather than lanosterol as the key intermediate. Unlike the tetracyclic lanosterol used in other kingdoms, PS biosynthesis begins with cycloartenol, the precursor molecule.^36^ This process occurs through three sequential stages: (i) generation of cycloartenol from acetyl-CoA, (ii) its conversion to 4-methyl-24-methylenelophenol, and (iii) the subsequent formation of β-sitosterol, stigmasterol, campesterol, and brassicasterol.^36^ The first two stages are universal to all PS biosynthesis. Cycloartenol acts as the primary branching point, with one metabolic route generating C24-alkyl PS and other leading to cholesterol synthesis. At the third stage, 4-methyl-24-methylenelophenol serves as a secondary branching point, directing synthesis toward either C24-methylated sterols, such as campesterol and brassicasterol, or C24-ethylated sterols, including β-sitosterol and stigmasterol.^36^ These unique branching mechanisms not only produce diverse sterols but also highlight the fundamental differences between PS biosynthesis and that of animal and fungi.

Some plants, such as tomatoes, potatoes, and diatoms, produce small amounts of cholesterol, primarily used in the synthesis of steroidal alkaloids and saponins. In plants, cholesterol synthesis uniquely begins with cycloartenol derived from 2,3-oxidosqualene and requires ten enzymatic steps.^37^ Unlike the animal pathway, this process includes a plant-specific reaction: Cyclopropyl sterol isomerase (CPI) catalyzes both the removal of the C-9 methyl group from 31-norcycloartanol, yielding 31-nor-24(25)-dihydrolanosterol, and the opening of the cyclopropane ring in cycloeucalyptol to form obtusifoliol.^38^ These CPI-mediated reactions represent critical rate-limiting steps in the PS biosynthetic pathway.

Although no lanosterol-based abbreviated sterol biosynthetic route has been identified in plants, accumulating evidence suggests that lanosterol retains biological functions in certain species. Notably, lanosterol metabolism occurs in *Euphorbia lathyrism* latex, where its biosynthesis has been documented.^39^^,^^40^ The conversion of cycloartenol to lanostane-type structures presents challenges for 4,4-dimethyl Δ^8^ sterol saponin production, as plant cycloeucalenol isomerases exhibit low catalytic efficiency toward 4,4-dimethyl cyclopropyl sterol substrates.^41^ These findings suggest the potential existence of LSS in plants. Supporting this hypothesis, genomic mining identified the *Arabidopsis thaliana* gene *At3g45130* as encoding the first LSS to be cloned from a plant.^42^ Further genomic analyses across various plant species indicate that LSS is broadly distributed among eudicotyledonous lineages. Intriguingly, plant LSS appears to have evolved independently, incorporating distinct catalytic motifs that differ from those in non-plant LSS enzymes, thereby ensuring precise product specificity. The sporadic occurrence of lanosterol and lanostane-type saponins in plants likely reflects the activity of this specialized LSS, rather than the promiscuous function of cycloartenol synthase.^42^ In summary, while plants primarily synthesize sterols through the cycloartenol pathway, they have also maintained an evolutionarily distinct lanosterol-derived metabolic branch with specialized metabolic roles.

### Fungal origin

**Figure 3.**
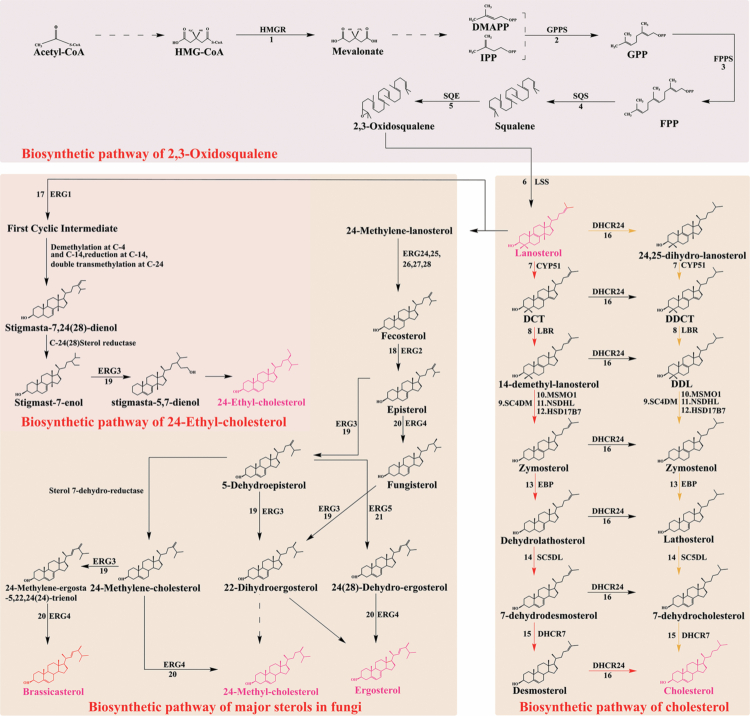
Transformation pathway of mycosterols. Lanosterol marks the first cyclic intermediate in the pathway of fungal sterol biosynthesis, and the biosynthesis of cholesterol in fungi follows the same route as in animals. In addition to cholesterol, several major sterols, such as ergosterol, 24-methyl cholesterol, 24-ethyl cholesterol, brassicasterol, and various intermediates, have been reported drawn with reference to 175 fungal species across the kingdom Fungi.^44^ Abbreviations: 1. HMGR, 3-hydroxy-3-methylglutaryl-CoA reductase; 2. GPPS, geranyl diphosphate synthase; 3. FPPS, farnesyl diphosphate synthase; 4. SQS, squalene synthase; 5. SQE, squalene epoxidase; 6. LSS, lanosterol synthase; 7. CYP51, sterol 14-demethylase; 8. LBR, δ(14)-sterol reductase; 9. SC4DM, sterol C-4 demethylation complex enzymes; 10. MSMO1, methylsterol monooxygenase 1; 11. NSDHL, sterol-4α-carboxylate 3-dehydrogenase; 12. HSD17B7, 3-keto-steroid reductase; 13. EBP, C-8 sterol isomerasee; 14.SC5DL, sterol-C5-desaturase; 15. DHCR7, 7-dehydrocholesterol reductase; 16. DHCR24, δ(24)-sterol reductase; 17. ERG1, squalene epoxidase; 18. ERG2, C-8 sterol isomerase; 19. ERG3, C-5 sterol desaturase; 20. ERG4, C-24(28) Sterol reductase; 21. ERG5, C-22 sterol desaturase; 22. ERG6, C-24 methyl transferase; ERG24, C-14 sterol reductase; ERG25, C-4 methyl oxidase; ERG26, C-3 steroldehydrogenaseic-4decarhox/ase; ERG27, 3-keto sterol reductase; ERG28, 3-keto sterol reductase; HMG-CoA, 3-hydroxy-3-methylglutaryl coenzyme A; IPP, isopentenyl pyrophosphate; DMAPP, dimethylallyl pyrophosphate; GPP, geranyl diphosphate; FPP, farnesyl pyrophosphate; DCT, 4,4-dimethyl-cholesta-8,14,24-trienol; DDCT, dihydro-4,4-dimethyl-cholesta-8,14,24-trienol; DDL:dihydro-14-dimethyl-lanosterol.

Mycosterols are sterols with a characteristic polycyclic structure consisting of four rings (three six-carbon and one five-carbon ring), and typically contain 27, 28, or 29 carbon atoms. Representative examples include cholesterol, ergosterol, and sitosterol-related compounds.^43^ C27 mycosterols, such as cholesterol, 22-dehydrocholesterol, and zymosterol, often feature unsaturated double bonds at the C-22 and C-24 positions. C28 sterols, including ergosterol and its derivatives,^44^ such as 22-dihydroergosterol,^45^ ergosta-5,7,24(28)-trienol,^46^ fecosterol,^47^ possesses a 28-carbon backbone. Sitosterol-related compounds, including sitosterol, 24-ethylcholesterol, and fucosterol, are characterized by a C29 backbone.^48^ Sterol composition varies among fungal taxa. For example, primitive fungi, such as those in the phylum *Chytridiomycota*, predominantly contain cholesterol. In contrast, more evolutionarily advanced groups, including *Ascomycota* and *Basidiomycota*, primarily synthesize ergosterol. Intermediate groups like the *Zygomycota* also primarily contain ergosterol, whereas others, such as *Mortierellaceae*, mainly produce 24-dehydrocholesterol.^49^ Among fungal species, the major sterol biosynthesis products include cholesterol, ergosterol, 24-methyl cholesterol, 24-ethyl cholesterol, and brassicasterol. Additionally, intermediates generated during the synthesis of 24-ethyl cholesterol represent a substantial fraction of mycosterols.^50^ Sterol biosynthesis in fungi involves a series of enzyme-catalyzed reactions, where intermediate metabolites sever as precursors for the generation of structurally diverse sterols (Figure 3).

As in animal cholesterol biosynthesis, lanosterol functions as the initial cyclic intermediate in the sterol biosynthetic pathway of fungi. The typical biosynthetic route for mycosterols involves methylation at the C-24 position of lanosterol, followed by demethylation and double-bond rearrangements at the C-4 and C-14 positions, a sequence that mirrors steps seen in cholesterol synthesis. These reactions ultimately result in the formation of common C28 sterols, which are prevalent in most fungal species. In higher plants, the C-24 ethyl group of sterols typically adopts the α-configuration, however, the exact configuration of this group in fungal sterols remains unclear.^51^ Ergosterol is the predominant sterol component of fungal cell membranes. Its biosynthesis differs from cholesterol biosynthesis in three key steps: (i) C-24 methylation, (ii) reduction at C-24(28), and (iii) insertion of a double bond at the C-22 position of the side chain.^52^^,^^53^ Therefore, although fungi and animals share a common sterol biosynthesis pathway centered on lanosterol, fungi employ distinct modification steps that result in the production of ergosterol. This sterol is structurally and functionally specialized to support the specific membrane composition and physiological needs of fungal cells.

**Figure 4.**
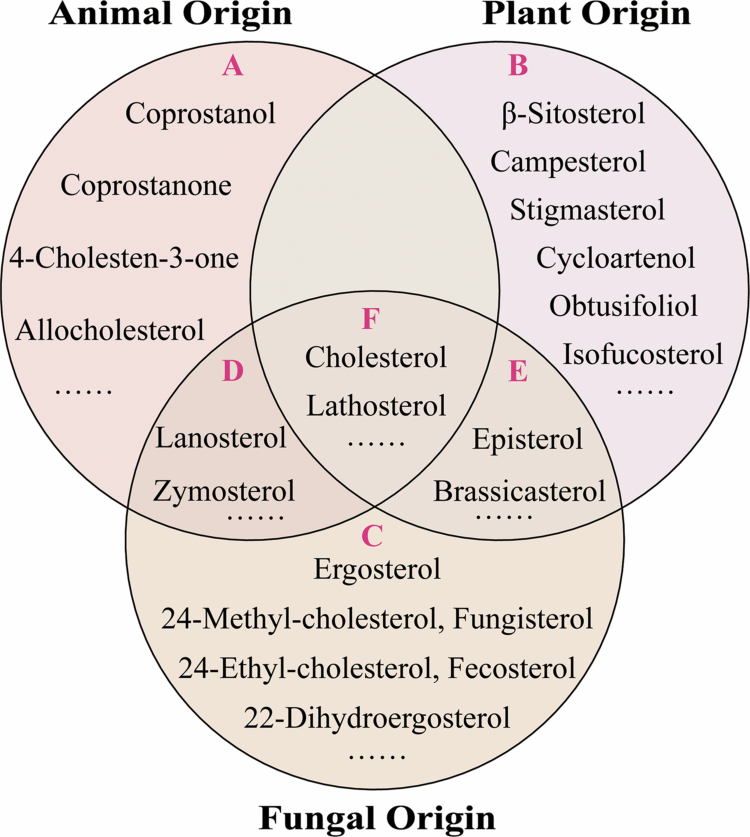
Schematic overview of sterol types and their biological sources. A, B, and C represent the unique sterol types found in animals, plants, and fungi, respectively. D represents the sterol types shared by animals and fungi. E represents the sterol types shared by plants and fungi. F represents the sterol types shared by animals, plants, and fungi.

Across animals, plants, and fungi, sterol synthesis initiates from acetyl-CoA and proceeds through a series of enzyme-catalyzed reactions to generate 2,3-oxidosqualene. In animals and fungi, 2,3-oxidosqualene is cyclized into lanosterol by LSS, followed by additional enzymatic modifications to ultimately produce cholesterol. However, PS metabolism in plants differs from that in animals and fungi. Instead of lanosterol, plants use cycloartenol synthase to convert 2,3-oxidosqualene into cycloartenol. Although plants are capable of synthesizing cholesterol, their biosynthetic route includes an additional enzymatic step not found in animals, catalyzed by CPI. This enzyme catalyzes the demethylation of 31-norcycloartanol at the C-9 position, producing 31-nor-24(25)-dihydrolanosterol. In animals, cholesterol can be further metabolized into coprostanol through enzymatic catalysis, a transformation that does not occur in plants or fungi. Taken together, there are both unique and shared sterol types in animals, plants, and fungi (Figure 4). Among them, cholesterol is the most abundant in animals,^6^ but it also exists in small amounts in plants and fungi. β-sitosterol, campesterol, and stigmasterol are the predominant sterols present in most plant species,^29^ while ergosterol is the primary sterol in many groups of fungi.^44^ Furthermore, the types of PS have the highest diversity, followed by fungi and then animals.^54^

## Sterol metabolism modulated by gut microbiota

**Figure 5.**
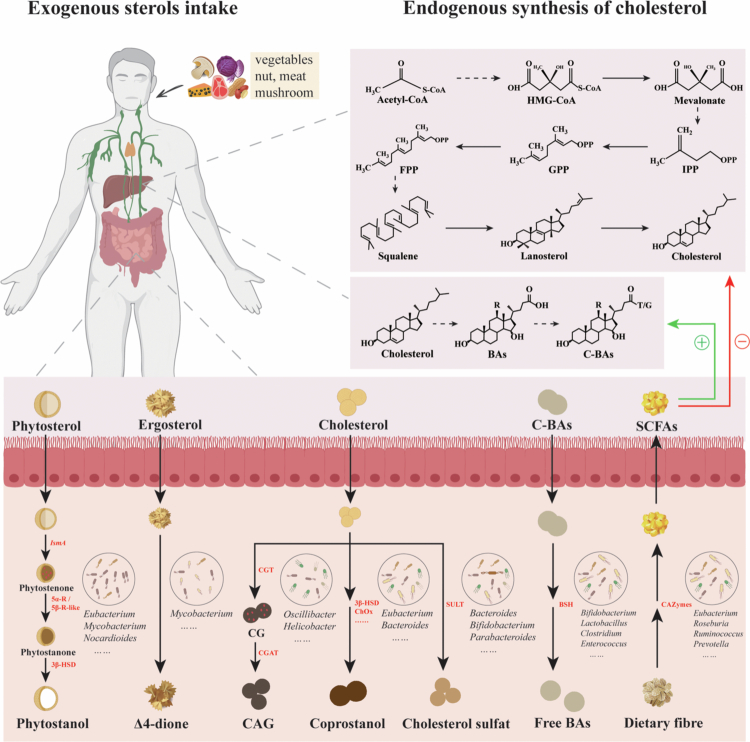
Metabolism of human sterol by gut microbiota. Humans obtain sterols from foods such as vegetables, nuts, meat, and mushrooms. Additionally, cholesterol is partly synthesized in the liver and transformed into BAs. Once in the intestine, sterols are metabolized by gut microbiota through multiple pathways. Confirmed enzymatic reactions are represented by solid lines, whereas hypothetical steps are indicated with dashed lines. Green arrows (+) indicate promotion, while red arrows (-) indicate inhibition. Abbreviations: HMG-CoA, 3-hydroxy-3-methylglutaryl coenzyme A; IPP, isopentenyl pyrophosphate; GPP, geranyl diphosphate; FPP, farnesyl pyrophosphate; BAs, bile acids; C-BA, conjugated bile acids; *IsmA*, Intestinal sterol metabolism A gene; 5α-R/5β-R-like, 5α-reductase/5β-reductase-like; 3β-HSD, 3β-hydroxysteroid dehydrogenases; **Δ**4-dione, androst-4-ene-3,17-dione; CGT, cholesterol α-glucosyltransferase; CG, cholesteryl α-D-glucopyranoside; CGAT, cholesteryl α-D-glucoside 6'-acyltransferase; CAG, cholesteryl 6′-O-acyl-α-D-glucopyranoside; ChOx, cholesterol oxidase; SULT, sulfotransferase; BSH, bile salt hydrolase; Free BAs, free bile acids; SCFAs, short-chain fatty acids; CAZymes, carbohydrate-active enzymes.

Currently research on the influence of gut microbiota on sterol metabolism has largely concentrated on cholesterol. Substantial evidence shows that gut bacteria participate in cholesterol metabolism through multiple mechanisms, including its transformation into coprostanol,^55^ cholesterol sulfate,^56^ and cholesteryl 6′-O-acyl-α-D-glucopyranoside (CAG),^57^ as well as indirectly via bile salt hydrolase (BSH) activity^58^ and the production of short-chain fatty acids (SCFAs).^59^ In contrast, the role of gut microbiota in metabolizing sterols derived from plants and fungi remains less understood. Nevertheless, emerging studies suggest that gut microbiota may participate in diverse metabolic pathways related to these sterol classes. Additionally, sterol-metabolizing enzymes encoded by gut-resident fungi are gradually recognized for their potential influence host sterol metabolism. Therefore, this section will explore the regulatory roles of gut microbiota in host sterol metabolism, with a particular emphasis on both bacterial and fungal perspectives (Figure 5). Furthermore, given the abundance of sterol-metabolizing enzymes encoded by the gut microbiota, we will propose potential pathways by which these microbes may participate in host sterol metabolism, informed by established sterol biosynthetic routes in plants and fungi.

### Bacterial pathways

With the rapid advancement of bioinformatics, the gut bacteria involved in sterol metabolism are increasingly being revealed. Since 1973, several gut bacterial strains capable of transforming cholesterol into coprostanol have been isolated. Kenny et al.^60^ identified a 3β-hydroxy-Δ5-steroid dehydrogenase (3β-HSD) from *Eubacterium coprostanoligenes* HL (ATCC 51222), originally isolated from a swine wastewater lagoon. This enzyme, encoded by the intestinal sterol metabolism A gene (*IsmA*), is a NADP^+^-dependent, oxygen-independent, cholesterol-induced HSD that catalyzes the conversion of cholesterol to 4-cholesten-3-one and of coprostanone to coprostanol. Notably, it does not utilize 3α-hydroxy BAs such as cholic acid or chenodeoxycholic acid as substrates.^61^ To date, this is the only known enzyme that directly converts cholesterol to coprostanol. Unfortunately, bacteria encoding *IsmA* homologs in the human gut remain uncultivable and have only been identified from metagenomic species (MSPs). For example, six *IsmA* homologs from uncultivated human gut microbiota were discovered through multiple correlation analyses of large-scale metagenomic and metabolomic datasets across several global cohorts.^60^ When heterologously expressed in *Escherichia coli*, lysates from all six homologs displayed activity in oxidizing cholesterol to 4-cholesten-3-one and converting coprostanol to coprostanone. By binning co-abundant genes into MSPs across more than 3000 datasets, 20 different MSPs containing *IsmA* genes were identified. They formed a coherent clade in the phylogenetic neighbourhood of *Clostridium* cluster IV, which contains health-associated species, including SCFAs producers. *IsmA*-encoding MSPs were relatively abundant (average 1.4%) in human metagenomes, with prevalence ranging from 37% to 92% of samples, which supports the idea that these bacteria are widespread constituents of the human gut microbiome.^60^ Eyssen et al.^62^ isolated *Eubacterium* sp. strain ATCC 21408 from rat cecal contents. This strain reduces the 5,6-double bond in cholesterol, yielding coprostanol as a 5β-saturated derivative. Another strain, *Eubacterium* 403, capable of converting cholesterol into coprostanol, was isolated from baboon feces.^28^ Both strains require plasmenylethanolamine for growth*.* In 2007*,* a human-derived strain, *Bacteroides* sp. strain D8, was isolated from human feces and shown to convert cholesterol into intermediate products such as 4-cholesten-3-one and coprostanone, and ultimately into coprostanol *in vitro*.^26^ Moreover, the biochemical pathway involved in the conversion of cholesterol and coprostanol into their respective sulfate derivatives has been elucidated. Cholesterol sulfation is mediated by microbial sulfotransferase (SULT), using 3′-phosphoadenosine-5′-phosphosulfate (PAPS) as a sulfur donor to form cholesterol-3-sulfate. In *Bacteroides thetaiotaomicron*, this transformation is catalyzed by the *Bt_0416* gene, and *Bt_0416*-like genes are commonly found in other *Bacteroidetes*.^56^^,^^63^ Yao et al.^56^ further identified homologs in *Firmicutes* and *Proteobacteria*, confirming SULT activity in *Parabacteroides merdae*. The substrate specificity of the SULT derived from *B. thetaiotaomicron* was found to depend on the steroid’s structure. This enzyme can also sulfate steroids with planar A/B rings, including isoallolithocholic acid, allolithocholic acid, isolithocholic acid, ergosterol, cholesterol, pregnenolone, β-sitosterol, lanosterol, and calcifediol. Additionally, *Helicobacter pylori* integrates cholesterol from host gastric epithelial cells into its membrane, where it is modified by α-glucosylation through the action of cholesterol *α*-glucosyltransferase (CGT), encoded by the *hp0421* gene.^64^ CGT attaches glucose to the 3-hydroxyl group of cholesterol, yielding cholesteryl α-D-glucopyranoside (CG). CG can subsequently be acylated by cholesteryl α-D-glucoside 6'-acyltransferase (CGAT), encoded by *hp0499*, to generate CAG. Using PROtein Sequence Embedding, Li et al.^65^ analyzed 41,934 proteins from *Oscillibacter* genome assemblies and identified candidates with structural similarity to CGT. Consequently, these findings highlight the expanding role of gut microbiota not only in cholesterol transformation and modification but also in enhancing the diversity and regulatory capacity of host sterol metabolism via distinct enzymatic mechanisms.

Within the liver, a portion of cholesterol is transformed into BAs, which are subsequently conjugated with glycine or taurine to generate conjugated bile acids (C-BAs) before being secreted into the small intestine. Some gut microbes produce BSH, which deconjugates C-BAs into free BAs. This increases the ratio of free BAs to C-BAs, promoting BAs excretion and consequently reducing the overall pools of both cholesterol and BAs.^66^ Approximately 26.03% of colonic bacteria possess BSH activity,^67^ predominantly among Gram-positive bacteria, including *Bifidobacterium*, *Lactobacillus*, *Clostridium*, *Enterococcus*, and *Listeria*,^68–70^ although BSH is also present in some Gram-negative bacteria, such as *Stenotrophomonas*, *Bacteroides*, and *Brucella*.^71^ In a study involving hamsters fed a high-cholesterol diet, supplementation with the SCFAs such as acetate (Ac), propionate (Pr), and butyrate (Bu) led to notable reductions in total cholesterol levels, by 24%, 18%, and 17%, respectively, indicating the cholesterol-lowering potential of SCFAs.^72^ Key contributors to Bu production in the human gut include species such as *Faecalibacterium prausnitzii, Eubacterium rectale, Eubacterium hallii, Roseburia intestinalis,* and *Ruminococcus bromii*. In addition, *Butyricimonas*, *Coprococcus*, *Dysosmobacter*, and *Subdoligranulum* also contribute significantly to Bu synthesis in the human gut.^72^^,^^73^ *Bacteroides* spp. are also prominent SCFA producers, with their abundance positively linked to fecal SCFA concentrations in various studies.^74^ Additionally, *Clostridium butyricum* has been identified as a significant producer of Bu, along with Ac and Pr.^75^ SCFAs suppress cholesterol biosynthesis by downregulating the expression of genes encoding enzymes that catalyze the conversion of acetyl-CoA to cholesterol.^76^ Gene expression profiling in human enterocytes demonstrated that both Pr and Bu downregulate nine genes implicated in cholesterol biosynthesis, including the gene encoding HMGR.^77^ Furthermore, SCFAs function as mediators in the transformation of cholesterol to BAs, further contributing to cholesterol reduction. Cholesterol 7α-hydroxylase (CYP7A1), the rate-limiting enzyme in BA biosynthesis, catalyzes the initial step in this conversion. Animal studies have shown that an SCFA mixture could boost *Cyp7a1* expression in hamster livers.^72^ This SCFA-induced upregulation enhances cholesterol catabolism into BAs and promotes cholesterol uptake from the circulation into the liver, without affecting intestinal absorption. This leads to greater BA excretion and a reduction in plasma cholesterol levels.

PS and mycosterols are not synthesized in the human body and must be acquired through dietary sources, such as from vegetables and mushrooms.^78^ These sterols influence the gut microbiota through several mechanisms. For example, high-dose supplementation with PS esters in mice significantly increased the relative abundance of *Bacteroidetes* and *Anaerostipes* in the intestinal microbiota.^79^ Moreover, ergosterol peroxide, a derivative of ergosterol, can be metabolized into cytotoxic compounds within the gastrointestinal tract of mice.^80^ These findings suggest that PS, mycosterols, and their metabolites may impact the composition and stability of the gut microbiota. Several gut bacterial species have been shown to mediate the microbial transformation of these sterols into bioactive intermediates. For example, *Mycolicibacterium neoaurum* VKM Ac-1815D, VKM Ac-1816D, and NRRL B-3805 have been reported to convert PS into androst-4-ene-3,17-dione (**Δ**4-dione) and androstadienedione.^81^^,^^82^ Wang et al.^83^ further identified several enzymes involved in PS metabolism by the gut microbiota. Among them, *Faecalibacterium prausnitzii* encodes 3-oxosteroid 1-dehydrogenase (EC 1.3.99.4), which is implicated in the degradation of β-sitosterol. Additionally, *Arthrobacter simplex* IAM 1660 has demonstrated the ability to convert campesterol, β-sitosterol, and stigmasterol into their corresponding intermediates—campest-4-en-3-one, stigmast-4-en-3-one, and stigmasta-4,22-dien-3-one, respectively.^84^ The genetics underlying sterol degradation, particularly for ergosterol, have been extensively studied in mycolic-acid rich actinobacteria, including *Mycobacterium (*e.g., *M. tuberculosis, M. smegmatis,* and *M. neoaurum), Rhodococcus*, *Gordonia* and other genera. Notably, *Mycobacterium* sp. BCS 396 converts ergosterol into 3-oxo-4,22-ergostadien-26-oic acid methyl ester, 3-oxo-1,4,22-ergostatrien-26-oic acid methyl ester, and 3-oxo-1,4,22-ergostatrien-26-oic acid, whose structures have been confirmed by IR, ^1^H NMR, ^13^C NMR, and mass spectroscopy.^85^ In contrast, relatively little is known about this process in *Nocardioides* and related actinobacteria.^86^ However, *Nocardioides simplex* could be able to utilize abundant PS as a carbon and energy source. For example, *N. simplex* VKM Ac-2033D can grow on PS, during which phytostenones are detected as major degradation intermediates.^86^ Furthermore, it has been proposed that in the intestinal tract, PS can undergo microbial conversion to intermediates like phytostenone and phytostanone, which are subsequently transformed into phytostanols.^87^ In this regard, sitosterol and campesterol are primarily converted into ethyl-coprostanol and methyl-coprostanol, which are then oxidized to form ethyl- and methyl-coprostanone, respectively.^88^ Although the microbial degradation pathway of stigmasterol remains largely unclear, ethylcoprostenol and ethylcoprostenone have been proposed as its degradation products.^89^ Preliminary research indicate that β-sitosterol is converted to ethylcoprostanol both *in vivo* and *in vitro*.^29^ In biotransformation experiments using a pure *Eubacterium* sp. culture obtained from rat feces, it was found that the microorganism targets the 5,6 double bond of β-sitosterol, campesterol, and stigmasterol, converting them into their 5β-saturated counterparts, known as phytostanones.^90^ Despite these advances, our understanding of the microbial metabolism of PS and mycosterols in the gut remains limited. Further research is needed to elucidate the precise microbial pathways and mechanisms responsible for their transformation.

### Eukaryotic pathways

Compared to bacteria, the fungal component of the gut microbiota is relatively limited, making up about 0.1% of the overall microbial population.^91^ The dominant fungal communities in the gut are classified within the phyla *Ascomycota*, *Basidiomycota*, and *Zygomycetes*,^92^^,^^93^ with the most abundant genera including *Candida*, *Saccharomyces*, *Penicillium*, *Aspergillus*, *Cryptococcus*, *Malassezia*, *Cladosporium*, *Galactomyces*, *Debaryomyces*, and *Trichosporon*.^94^^,^^95^ It is relatively uncommon for fungi to have a direct function in cholesterol metabolism within the gut. In the early stages of cholesterol biosynthesis, inhibition of HMGR leads to the accumulation of HMG-CoA, which is further degraded into simpler compounds, bypassing the production of sterol ring-containing lipophilic intermediates.^96^ Statins, a class of polyketide-structured molecules derived from fungal secondary metabolism, inhibit HMGR activity and thus regulate cholesterol synthesis.^97^ In yeast, treatment with lovastatin at doses of 40–50 μg/mL can suppress the synthesis of squalene, oxysterols, and ergosterol.^98^ In addition, β-sitosterol is converted to stigmasterol by the action of sterol−22-desaturase and ∆5,7campesterol is also converted to ergosterol through the action of the same enzyme.^99^ *Monascus* spp., commonly known as red yeast mold, have been identified as crucial regulators of cholesterol metabolism in the gut. Endo et al.^100^ isolated Monacolin K from *Monascus ruber*, a compound structurally analogous to HMG-CoA. Monacolin K competes with HMG-CoA for binding to HMGR, thereby disrupting the formation of mevalonate, a pivotal molecule in the cholesterol biosynthetic pathway, and subsequently limiting cholesterol synthesis.

The genus *Candida* possesses sterol 14α-demethylase (CYP51), an essential enzyme for ergosterol biosynthesis. CYP51, the only cytochrome P450 enzyme involved in sterol synthesis, catalyzes the initial demethylation step following sterol ring cyclization.^101^ Another crucial enzyme, C-5 sterol desaturase, introduces a double bond at the C-5,6 position of saturated sterols such as episterol and ergosta-7,22-dien-3β-ol, acting near the end of the ergosterol synthetic pathway to produce ergosta-5,7,22-trien-3β-ol.^102^ The *Mucor lusitanicus* genome encodes three sterol 24-C-methyltransferase enzymes, Erg6a, Erg6b, and Erg6c. Erg6 catalyzes the methylation at the C-24 position of zymosterol to form fecosterol and also contributes to the conversion of lanosterol to eburicol during ergosterol biosynthesis.^103^ Among these, Erg6b plays a particularly critical role in the final steps of ergosterol biosynthesis, knocking out this gene greatly lowers ergosterol content and results in the accumulation of zymosterol.^103^ Similarly, the absence of Erg6 impairs the growth of *Cryptococcus neoformans* at 30 and 35 °C,^104^ a phenotype also found in *Candida lusitaniae*. *Saccharomyces* genus (e.g., *Saccharomyces cerevisiae*, commonly known as brewer's yeast) encode multiple key enzymes for sterol biosynthesis and regulation. SQS activity, in particular, is tightly controlled.^105^ M'Baya et al.^106^ show that SQS activity rises fivefold in yeast sterol auxotrophs facing sterol limitation, while it is repressed in wild-type strains grown anaerobically with sterol excess. Moreover, *Limosilactobacillus fermentum* has been shown to inhibit the growth of *Candida glabrata* by consuming ergosterol.^107^ Mailänder-Sánchez et al.^108^ also reported similar results, showing that coculture of *C. albicans* with *Lactobacillus rhamnosus* GG suppressed ergosterol biosynthesis and reduced the cellular ergosterol content in the yeast. These findings underscore the potential of certain fungal species to modulate host sterol metabolism and highlight their promising applications in metabolic regulation.

Based on macrogenomic/macrotranscriptomic analyses, functional assays and gene validation have revealed novel enzymes and biosynthetic routes beyond the canonical sterol pathways. One of the best examples is the widespread occurrence of cycloartenol, and cycloartenol synthase (CAS), in prokaryotes and eukaryotes like *Dictyostelium discoidum* or *Naegleria*.^109^ Bacteria belonging to the genera *Gordonia*, *Nocardia* and *Rhodococcus* are able to degrade the side chains of PS.^110^^,^^111^ *Stigmatella aurantiaca* is a myxobacterium that produce sterols from cycloartenol.^112^ High-throughput transcriptome sequencing of *Mycobacterium* sp. VKM Ac-1817D grown with or without PS revealed significant upregulation of 260 genes in the presence of PS, many of which are involved in steroid catabolism. Among these, two of the five genes encoding the oxygenase unit of 3-ketosteroid-9α-hydroxylase (kshA) were upregulated by 55- and 25-fold, respectively, while one of the two genes encoding its reductase subunit (kshB) was upregulated 40-fold.^113^ Metagenomic studies further identified numerous ergosterol synthesis genes (e.g., C-22 sterol desaturase, cytochrome p450, and lanosterol 14α-demethylase) within the *Ascomycota phylum*.^114^ In *Sarocladium terricola*, transcriptomic analysis demonstrated significant upregulation of ergosterol synthesis genes *ERG3*, *ERG5*, and *ERG25* when cultured in potato glucose medium. Moreover, recent study identified *Erg29* as a participate in the methyl sterol oxidase step of ergosterol biosynthesis, with genome-wide expression data showing that *ERG* gene expression is altered in response to iron deficiency.^115^ The growing accessibility to genome mining is rapidly broadening our understanding of the origin and distribution of sterol biosynthetic capabilities across the tree of life.^116^ Nonetheless, previously unknown genes and enzymes are likely to be discovered through forward genetics and serendipitous genetic screenings. Interestingly, even minor variations in enzyme sequences like single amino acid substitutions, can redirect biosynthetic pathways, for instance, a mutation in cycloartenol synthase can convert its function into that of LSS.^117^ Looking ahead, large-scale genomic and metagenomic analyses, coupled with targeted functional validation assays, will be crucial for uncovering novel sterol biosynthetic enzymes, alternative pathways, and new dimensions of cellular homeostasis.

## Sterol metabolism related diseases mediated by gut microbiota

**Figure 6.**
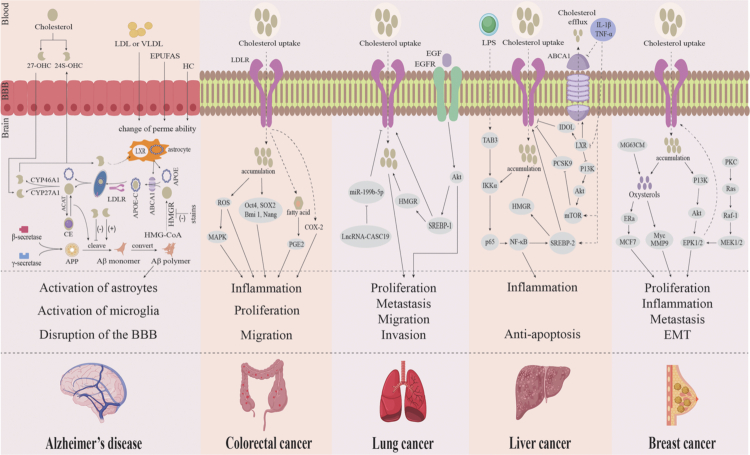
Sterol metabolism disorders and disease. (i) Cholesterol binds to APOE to form APOE-C particles, which are secreted into the extracellular fluid with the assistance of ATP-binding cassette transporter A1. These APOE-C particles are then endocytosed by neurons via the LDLR. Inside the neuron, APOE-C dissociates, LDLR is recycled back to the cell membrane, and cholesterol is metabolized through three pathways, contributing to APP metabolism and Aβ production. (ii) LDLR-driven pathways in cancer cells.^120^ (1) In colorectal cancer, cholesterol accumulation promotes tumor progression by activating the ROS/MAPK signaling pathway and upregulating key stemness-related genes, including *Sox2*, *Oct4*, *Nanog* and *Bmi1*. (2) In lung cancer, activation of the EGFR/Akt/SREBP−1/LDLR signaling pathway contributes to tumor progression. (3) In liver cancer, inflammatory cytokines such as IL-1β and TNF-α reduce LDLR degradation by inhibiting the LXR/IDOL and PCSK9 pathways, while simultaneously enhancing LDLR expression via upregulation of SREBP−2. Additionally, LPS activates the NF-κB pathway, which increases the expression of SREBP−2, HMGR, and LDLR, leading to cholesterol accumulation. This buildup further stimulates the NF-κB pathway, creating a positive feedback loop that amplifies inflammation. (4) In breast cancer, cholesterol accumulation triggers the PI3K/Akt/ERK1/2 pathway along with its conversion to oxysterols, which further activates c-Myc and EMT to accelerate the proliferation and metastasis of breast cancer cells. Abbreviations: 27-OHC, 27-hydroxycholesterol; 24S-OHC, 24S-hydroxylcholesterol; LDL, low-density lipoprotein; VLDL, very low density lipoprotein; EPUFAs, essential polyunsaturated fatty acids; HC, hypercholesterolemic; BBB, blood–brain barrier; LXR, liver X receptors; APOE, apolipoprotein E; HMGR, 3-hydroxy-3-methylglutaryl-CoA reductase; ABCA1, ATP-binding cassette transporter A1; APOE-C, apolipoprotein E cholesterol; LDLR, low density lipoprotein receptor; CYP46A1, 24S-hydroxylase; CYP27A, cholesterol 27α-hydroxylase; ACAT, acyl-CoA cholesterol acyltransferase; CE, cholesterol ester; APP, amyloid protein precursor; Aβ, amyloid beta; EGF, epidermal growth factor; EGFR, epidermal growth factor receptor; LPS, lipopolysaccharides; EMT, epithelial-mesenchymal transition.

As described in Section 3, gut microbiota exert significant effects on host sterol metabolism. Microbiota-associated sterol metabolites, such as cholesterol, sterol intermediates, and oxysterols, have been associated with the onset and progression of various diseases, particularly AD and cancer.^118^^,^^119^ Therefore, this review evaluates the role microbiota-associated sterol metabolites in modulating AD and cancer, with a focus on the underlying molecular mechanisms (Figure 6).

### Sterol metabolism disorders and Alzheimer's disease

Gut microbiota significantly contribute to both the synthesis and metabolism of brain sterols by generating a range of metabolic byproducts. These alterations subsequently affect cerebral cholesterol levels, thereby influencing cognitive function and emotional regulation. For example, metabolites produced by the gut microbiota such as BAs serve as signaling molecules that can traverse the gut-brain axis and modulate cholesterol metabolism by activating the farnesoid X receptor (FXR) and membrane-bound Takeda G protein-coupled receptor (TGR5).^121^ The brain contains the highest cholesterol content in the body, predominantly in a free, non-esterified form. It is primarily localized in myelin, astrocytes, and neuronal membranes, where it plays vital roles in neuronal development, synaptic plasticity, and overall brain function.^122^ Given these critical roles, disturbances in brain cholesterol metabolism can profoundly impair brain function. Under hypercholesterolemic (HC) conditions, the integrity of the blood-brain barrier (BBB) is compromised, allowing peripheral cholesterols to infiltrate the brain. This disruption facilitates the entry of essential polyunsaturated fatty acids (EPUFAs), low-density lipoproteins (LDLs), and very low density lipoproteins (VLDLs), leading to dysregulated brain cholesterol metabolism.^123^ Specifically, elevated cholesterol levels contribute to the pathogenesis of AD by elevating membrane cholesterol, which facilitates the binding of amyloid protein precursor (APP) to lipid rafts.^124^ Furthermore, elevated cellular cholesterol boosts the catalytic activity of both β-secretase and γ-secretase, leading to increased Aβ production.^125^ Higher cholesterol levels also increase Aβ production and promote the transformation of soluble, non-toxic, helix-rich Aβ monomers into aggregated, β-sheet-rich toxic forms, a critical event in AD progression. Moreover, cholesterol esterification is associated with elevated Aβ synthesis. Conversely, inhibition of acyl-CoA cholesterol acyltransferase (ACAT) reduces the generation of cholesterol esters (CEs), thereby suppressing Aβ generation.^126^ This suggests that the balance between free cholesterol and CE also affects Aβ production.

In the brain, the main oxysterols, 24S-hydroxycholesterol (24S-OHC) and 27-hydroxycholesterol (27-OHC), are oxidation products of cholesterol. These are produced via the catalytic actions of cholesterol 24S-hydroxylase (CYP46A1) and cholesterol 27α-hydroxylase (CYP27A1), respectively.^123^ Neuron-derived 24S-OHC enters the bloodstream via the blood-brain barrier (BBB) and is eventually processed by the liver, helping maintain cholesterol homeostasis in the brain. A portion of 24S-OHC is taken up by astrocytes, where it activates liver X receptors (LXRs), promoting the expression of apolipoprotein E (APOE) and ATP-binding cassette transporter A1 (ABCA1).^123^ Fecal 16S rDNA sequencing and taxonomic profiling have revealed reduced abundance of *Roseburia* and lower SCFA levels in mice exposed to 27-OHC.^127^ Multiple studies have demonstrated that oxysterols play an essential role in modulating Aβ production. In particular, the balance between 24S-OHC and 27-OHC significantly impacts the development and progression of AD.^128^ A higher ratio of 27-OHC to 24S-OHC correlates with increased Aβ synthesis.^123^ For example, SH-SY5Y cells treated with 27-OHC is associated with higher levels of β-secretase and Aβ. Furthermore, 27-OHC levels in the brain are mainly regulated by BBB permeability and structural soundness. When the BBB is compromised, substantial amounts of 27-OHC can enter from the bloodstream. Conversely, 24S-OHC increases neuronal α-secretase activity and inhibits the activity of β-secretase,^129^ thereby decreasing Aβ production. Supporting this, Heverin et al.^130^ reported decreased 24S-OHC and elevated 27-OHC levels in the brains of AD patients. These findings suggest that targeting cholesterol metabolism may be an effective strategy for preventing and treating AD. Current interventions for controlling cholesterol include extended-release niacin, cholesterol absorption inhibitors, ACAT inhibitors, and cholesteryl ester transfer protein inhibitors.^131–133^ In the future, integrating such pharmacotherapies with probiotics may offer synergistic lipid-lowering effects, thereby enabling dose reduction and minimizing potential drug-related adverse effects.

### Sterol metabolism disorders and cancer

Gut microbiota, particularly commensal *Clostridia*, can enhance host cholesterol synthesis through metabolites such as branched-chain amino acids (BCAAs), which are known to increase the proliferation and stem-like properties of colorectal cancer cells.^134^ In over 60% of melanoma patients, increased expression of multiple cholesterol biosynthetic genes has been noted.^135^ Additionally, higher serum cholesterol levels are linked to an increased risk of several cancers, including colorectal, lung, and liver cancers.^136–138^ The low-density lipoprotein receptor (LDLR) functions as a transmembrane receptor critical for cholesterol uptake and cellular cholesterol regulation.^120^ Gut microbiota-derived SCFAs, particularly Bu, modulate the activity of sterol regulatory element-binding protein 2 (SREBP−2), which boosts LDLR expression and enhances LDL uptake, ultimately lowering circulating LDL cholesterol (LDL-C) levels.^139^ However, recent studies have revealed significant variations in LDLR expression between cancer cells and normal cells. While tightly regulate cholesterol homeostasis by balancing extracellular LDLs uptake and de novo cholesterol synthesis, this regulatory feedback loop is often disrupted in cancer cells.^140^ As a result, cancer cells often exhibit increased LDLR expression and enhanced uptake of LDL-derived cholesterol that fuels tumor growth.

In colorectal cancer, especially at advanced N or M stages, LDLR expression is notably increased. Wang et al.^141^ showed that LDL/cholesterol promotes the progression from intestinal inflammation to colorectal cancer, potentially mediated by ROS activation and MAPK signaling pathways. LDL was found to enhances colorectal cancer cell migration and upregulates stemness-related genes, including SRY-related high-mobility group box 2 (Sox2), octamer-binding transcription factor 4 (Oct4), Nanog, and B-lymphoma Mo-MLV insertion region 1 (Bmi1). Tanaka et al.^142^ identified that LDLR activation supports prostaglandin E_2_ (PGE_2_) production via the metabolism of fatty acids in the COX pathway. Furthermore, LDLR may facilitate inflammation by influencing the production of pro-inflammatory cytokines such as ROS, COX−2, and PGE_2_. This mechanism promotes the abnormal proliferation of colorectal cancer cells in a persistently inflamed microenvironment, thus contributing to colorectal cancer progression.^143^ Gueddari et al.^144^ observed a significant increase in LDLR expression in the human lung adenocarcinoma cell line A549 compared to normal lung epithelial cells. Qu et al.^145^ demonstrated that elevated levels of LncRNA CASC19 promote the proliferation, migration, and penetration of non-small-cell lung cancer (NSCLC). Further studies revealed that CASC19 contributes to NSCLC progression by regulating the miR-301b-3p/LDLR interaction, leading to increased LDLR expression and enhanced tumor cell growth and metastasis.^146^ Prolonged chronic inflammation is known to drive liver fibrosis and the onset of hepatocellular carcinoma.^147^ LDLR enhances the HCV-E2 protein-induced activation of the MAPK/ERK pathway, which in turn promotes the growth of Huh−7 hepatoma cells.^148^ As mentioned above, LDLR, a key regulatory factor in cholesterol metabolism, has demonstrated considerable pathogenic potential in the onset and development of various cancers. By mediating inflammatory responses, maintaining cancer stemness, and activating multiple pro-proliferative signaling pathways, LDLR has emerged as a central hub linking metabolic reprogramming to malignant tumor evolution. Given its extensive involvement in solid tumors like colorectal, lung, and liver cancer, LDLR presents a promising therapeutic target and prognostic biomarker, deserving further exploration and integration into future precision oncology strategies.

Oxysterols exert pro-carcinogenic effects in lung cancer by modulating inflammatory pathways and interacting with oxysterol-binding proteins.^149^ *In vitro* studies, Chen et al.^150^ revealed that 25-hydroxycholesterol (25-HC) enhances migration and invasion of lung adenocarcinoma cells. In colorectal cancer, 27-OHC accumulates pathologically in advanced stages and promotes disease progression. Rossin et al.^151^ observed increased 27-OHC levels in advanced-stage colorectal cancer, suggesting its potential role in promoting cancer cell survival and infiltration. Warns and colleagues^152^ further demonstrated that treatment of SW620 and Caco−2 cells with 27-OHC reduced cell growth without inducing cytotoxicity or apoptosis, implicating 27-OHC in altered cellular differentiation via modulation of the protein Schlafen Family Member 12. In addition, oxysterols may play a role in breast cancer pathology and progression. For example, oxysterols secreted by osteoblast-like MG63CM cells promoted the migration of MCF7 and MDA-MB−231 breast cancer cells, suggesting a contribution to bone metastasis formation in breast cancer patients.^153^ Moreover, exposure of MCF7 cells to 27-OHC reduced E-cadherin and β-catenin expression, indicating a role for 27-OHC in epithelial-mesenchymal transition.^154^ Beyond promoting breast cancer progression, oxysterols are suspected to interfere with hormonal therapies. Specifically, 25-HC enhanced proliferation of MCF7 cells via the induction of ERa (ESR1) target genes.^155^ Oxysterols also act alongside ROS and lipid peroxides to induce metabolic disturbances, impair issues repair, and cause DNA damage, thereby contributing to cholangiocarcinoma formation.^156^ Taken together, oxysterols play diverse roles in cellular physiology and pathology, influencing not only disease progression but also the effectiveness of cancer therapies. They may also hold promise as biomarkers. To fully elucidate their role in carcinogenesis and their potential in cancer prevention or treatment, further comprehensive investigations are needed. Such research should integrate genomic, proteomic, and metabolomic profiling of both circulating and tissue-specific samples from patients with various pathologies to clarify the clinical utility of oxysterols in diagnosis and therapy.

## Sterol metabolism: from a disrupted equilibrium to dietary opportunities

**Figure 7.**
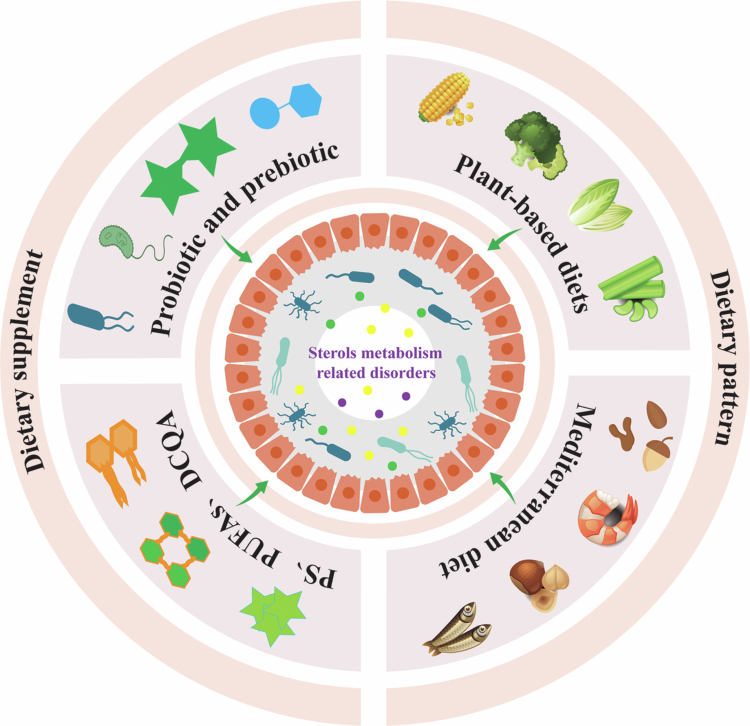
Sterol metabolism: From a disrupted equilibrium to dietary opportunities. Dietary components, probiotic supplements, and certain dietary patterns including plant-based and Mediterranean diets may serve as potential therapeutic strategies for sterol metabolism disorders. Abbreviations: PS, polysaccharides; PUFAs, polyunsaturated fatty acids; DCQA, dicaffeoylquinic acid.

Diet significantly influences both the composition and function of the gut microbiota.^157^ It not only affects host metabolism directly through specific functional components but also indirectly modulates sterol metabolism by shaping the structure and metabolic activity of the gut microbiota.^158^^,^^159^ This dual function highlights the importance of diet in preventing and managing disease progression. Dietary components, probiotic supplementation, and dietary patterns have all demonstrated significant effects in this regulatory process (Figure 7).

Polysaccharides are key dietary components that regulate sterol metabolism primarily by modulating the gut microbiota. Indigestible polysaccharides that reach the colon are fermented by members of *Bacteroidetes* and *Firmicutes* into SCFAs such as Ac, Pr, and Bu, which subsequently participate in cholesterol metabolism.^160^^,^^161^ For example, seaweed polysaccharides not only promote the proliferation of Bu-producing bacteria such as *F. prausnitzii* but also enhance BAs synthesis by increasing BSH activity and *Cyp7a1* expression, thereby reducing cholesterol levels.^162–165^ Similarly, fungal-derived polysaccharides, such as those from *Auricularia*, enhance the proliferation of bacteria such as *Oscillibacter* and *Lactobacillus*, which in turn boost SCFA production and help lower cholesterol.^166^ Polyunsaturated fatty acids (PUFAs), especially omega−3 fatty acids, indirectly influence cholesterol metabolism by promoting the proliferation of beneficial gut microbes like *Lachnospiraceae* and *Bifidobacterium*.^167^^,^^168^ Moreover, dietary sources such as walnuts and fish oil (rich in omega−3 PUFAs) have been found to increase the abundance of *Prevotella*, a genus linked to increased BA synthesis and lower serum cholesterol levels.^169^ Plant-derived dicaffeoylquinic acid (DCQA), a natural antioxidant, also plays a key role in reshaping the gut microbiota composition, particularly through the selective stimulation of beneficial bacteria, including *Bifidobacterium* and *Akkermansia*, thereby enhancing BA metabolism and cholesterol regulation.^170^ Probiotics, including *Bifidobacterium* and *Lactobacillus*, help reduce the reabsorption of intestinal BAs by increasing BSH activity, which promotes BA deconjugation.^171^ These probiotics also ferment dietary fibers and other substrates to produce SCFAs, including Ac, Pr, and Bu. Among them, Pr inhibits HMGR activity in the liver, thereby reducing cholesterol biosynthesis.^172^ In addition to regulating cholesterol homeostasis, dietary interventions have also been observed to modulate the metabolism of PS and mycosterols through the gut microbiota. For instance, galactooligosaccharides were found to increase the levels of β-sitosterol, sitosterol, campesterol, campestanol, and stigmasterol during dynamic gastrointestinal and colonic fermentation, accompanied by an increased abundance of the *Parabacteroides* genus and the *Synergistaceae* and *Lachnospiraceae* families.^173^ Concurrently, our previous *in vivo* experiments showed that polysaccharides from *Cordyceps militaris* intake in mice significantly increased brassicasterol levels through modulation of the gut microbiota.^174^ In addition, *Limosilactobacillus fermentum* and *Lactobacillus rhamnosus* GG were shown to regulate the ergosterol synthesis by interacting with the intestinal *Candida*.^107^^,^^108^

In addition to specific functional components and probiotic supplementation, dietary patterns, including plant-based and Mediterranean diets (MedDiet), are also vital in shaping gut microbiota composition and regulating cholesterol metabolism. Plant-based diets are characterized by minimal animal food consumption,^175^ and are rich in bioactive compounds, including dietary fiber, PS, phenolics, carotenoids, flavonoids, and saponins, mainly sourced from whole grains, fruits, vegetables, and legumes.^176^ A meta-analysis by Koch et al.^177^ found that, compared to omnivorous diets, plant-based diets significantly reduced total cholesterol by 0.34 mmol/L, LDL-C by 0.30 mmol/L, and apolipoprotein B by 12.92 mg/dL, equivalent to relative reductions of 7%, 10%, and 14%, respectively. These effects represent approximately one-third of the cholesterol-lowering efficacy typically achieved with statin therapy. The MedDiet, as described by Ancel Keys in the 1960s, is low in saturated fats and high in vegetable oils, reflecting dietary habits in Greece and Southern Italy. It is particularly rich in the marine omega−3 PUFAs, extensively studied for their cardiovascular benefits.^178^ Meslier and colleagues^179^ showed that adopting the MedDiet reduced total plasma cholesterol within just four weeks. Metabolomic analysis of feces, urine, and blood revealed distinct biomarker changes post-intervention, such as increased levels of urolithins, tryptophan betaine, and oxindole−3-acetic acid, alongside decreased levels of carnitine, p-cresol, and indoxyl sulfate, potential indicators of MedDiet adherence. Furthermore, the composition of the gut microbiota shifted, with increased abundance of *Faecalibacterium prausnitzii* and *Roseburia*, and reduced presence of *Ruminococcus gnavus* and *R. torques*. To sum up, dietary patterns rich in dietary fiber, polysaccharides, PUFAs, and plant-derived bioactive molecules have been proven to influence cholesterol metabolism by modulating the gut microbiota, thus supporting cholesterol balance. Unlike traditional pharmacological interventions, nutrition-based modulation of the gut microbiota provides a gentler, more systemic approach with potentially fewer side effects.

In the future, cholesterol management strategies are expected to adopt a dual-track approach. On one hand, there is a growing focus on systematically investigating how polysaccharides, fatty acids, and phytochemicals regulate the gut microbiota. To clarify the complex relationship between microbiota-derived metabolites and sterol metabolic pathways, originating from animals, plants, and fungi, the integration of multi-omics technologies including metagenomics, metatranscriptomics, metaproteomics, metabolomics, culturomics, and isotope techniques^180^ has become crucial. Meanwhile, several databases have been established to support this research, such as FoodDB and the USDA food composition databases, as well as the Virtual Metabolic Human and AGORA2. These resources integrate the metabolic capabilities of food, humans, and bacteria, offering predictive models that improve our understanding of the interactions among dietary nutrients, microbiota composition, sterol sources, and human health.^181^ On the other side, the integration of precision nutrition with individual gut microbiome profiles is paving the way for personalized dietary interventions. Within the framework of Industry 4.0, artificial intelligence is becoming a key driver of innovation in this field, enhancing both the efficiency and speed of digital nutrition assessments.^182^ For example, Liang et al.^183^ developed a machine learning (ML) algorithm capable of accurately predicting individual postprandial glucose responses, enabling the design of personalized dietary interventions for diabetes patients. Similarly, Kanegae et al.^184^ designed a highly accurate ML-based prediction model for identifying normotensive individuals at a risk of developing hypertension, allowing for early, non-pharmacological interventions. Alfonsi et al.^185^ introduced a mobile application, iSpy, which assists adolescents with type 1 diabetes in estimating carbohydrate intake, thereby supporting better dietary management. In the context of gut microbiota-mediated sterol metabolism and related diseases, ML algorithms such as regularized logistic regression, random forest, and XGBoost can be employed alongside clinical factors, dietary habits, and microbiome features to develop intelligent nutritional tools. Such tools could accurately assess daily nutrient intake, including macronutrients and micronutrients such as retinol, vitamins D and E, thiamine, calcium, sodium, iron, selenium, copper, and zinc, for patients with cancer or neurodegenerative diseases, ultimately guiding tailored nutritional strategies and dietary recommendations.

## Concluding remarks

Sterol metabolism is a complex physiological process co-regulated by interactions between the host and gut microbiota. In this review, we provide a summary of the biological pathways involved in sterol metabolism across different sources (animals, plants, and fungi), discuss recent advances in our understanding of how the gut microbiota regulates sterol metabolism, and elaborate on the underlying mechanisms by which these processes are implicated in AD and cancer. Furthermore, we highlight the potential of diet-based interventions in regulating sterol metabolism. However, much remains to be explored for a fuller understanding of the complex interplay of sterols with the host and gut microbiota, (i) the metabolic pathways of PS and ergosterol in the gut remain poorly understood and require further investigation, particularly regarding their microbial transformation within the gastrointestinal tract and their contributions to health and disease; (ii) integration of multi-omics technologies should be utilized to identify strains and enzymes in the gut associated with sterol metabolism; and (iii) large-scale, longitudinal population cohorts should be established and combined with wearable devices, digital health applications, and monitoring equipment to capture comprehensive datasets on dietary habits, microbiome profiles, metabolomics, and health indicators. Such integrative datasets will enable the identification of dietary/drug biomarkers and facilitate the development of microbiota-targeted therapies for sterol metabolism disorders. By bridging microbiological insights with clinical perspectives, future research holds promise for deepening our understanding of host-microbiota-sterol interactions and for driving therapeutic innovation.

## Data Availability

There is no research data in this review.
